# Magnetic Resonance Imaging-Based Radiomics for the Prediction of Progression-Free Survival in Patients with Nasopharyngeal Carcinoma: A Systematic Review and Meta-Analysis

**DOI:** 10.3390/cancers14030653

**Published:** 2022-01-27

**Authors:** Sangyun Lee, Yangsean Choi, Min-Kook Seo, Jinhee Jang, Na-Young Shin, Kook-Jin Ahn, Bum-soo Kim

**Affiliations:** Department of Radiology, Seoul St. Mary’s Hospital, College of Medicine, The Catholic University of Korea, Seoul 06591, Korea; 22000472@cmcnu.or.kr (S.L.); 21600406@cmcnu.or.kr (M.-K.S.); znee@catholic.ac.kr (J.J.); nyshin@catholic.ac.kr (N.-Y.S.); ahn-kj@catholic.ac.kr (K.-J.A.); bumrad@catholic.ac.kr (B.-s.K.)

**Keywords:** nasopharyngeal carcinoma, radiomics, survival, meta-analysis

## Abstract

**Simple Summary:**

More than 70% of patients with nasopharyngeal carcinoma (NPC) present with a locoregionally advanced state. Although the initial staging of NPC is primarily based on TNM staging, there is currently no well-established prognostic marker for NPC. Recently, radiomics has received considerable research attention as a potential prognostic biomarker for NPC. The aim of this systematic review and meta-analysis was to comprehensively evaluate the prognostic value of pretreatment magnetic resonance imaging (MRI)-based radiomics for NPC. The analyzed radiomic models demonstrated modest prognostic values, with a pooled mean estimated Harrell’s concordance index (C index) of 0.762. The prognostic models developed using more than eight radiomic features had significantly higher C-indices than those developed using fewer features. Our findings provide evidence that MRI-based radiomics may have a modest prognostic role in the treatment of NPC. However, more consistent study protocols are needed to verify the generalizability of radiomics.

**Abstract:**

Advanced non-metastatic nasopharyngeal carcinoma (NPC) has variable treatment outcomes. However, there are no prognostic biomarkers for identifying high-risk patients with NPC. The aim of this systematic review and meta-analysis was to comprehensively assess the prognostic value of magnetic resonance imaging (MRI)-based radiomics for untreated NPC. The PubMed-Medline and EMBASE databases were searched for relevant articles published up to 12 August 2021. The Transparent Reporting of a Multivariable Prediction Model for Individual Prognosis or Diagnosis (TRIPOD) checklist was used to determine the qualities of the selected studies. Random-effects modeling was used to calculate the pooled estimates of Harrell’s concordance index (C-index) for progression-free survival (PFS). Between-study heterogeneity was evaluated using Higgins’ inconsistency index (*I*^2^). Among the studies reported in the 57 articles screened, 10 with 3458 patients were eligible for qualitative and quantitative data syntheses. The mean adherence rate to the TRIPOD checklist was 68.6 ± 7.1%. The pooled estimate of the C-index was 0.762 (95% confidence interval, 0.687–0.837). Substantial between-study heterogeneity was observed (*I*^2^ = 89.2%). Overall, MRI-based radiomics shows good prognostic performance in predicting the PFS of patients with untreated NPC. However, more consistent and robust study protocols are necessary to validate the prognostic role of radiomics for NPC.

## 1. Introduction

Nasopharyngeal carcinoma (NPC) is an endemic cancer in Southeast Asia and Southern China, with an annual incidence rate of 50–80 patients per 1,000,000 population [1]. However, its annual incidence rate in Europe is relatively low at 4.7 patients per 1,000,000 population [2]. The standard treatment strategy for NPC involves concurrent chemoradiation therapy (CCRT) with or without adjuvant chemotherapy [3]. The prognosis of patients treated with CCRT is relatively fair, with a five-year overall survival and progression-free survival (PFS) rate of approximately 72% [4]. 

Initial cancer staging for NPC is primarily based on TNM staging according to the 8th edition of the American Joint Committee on Cancer guidelines [5]. Although TNM staging is currently the gold standard for the prognostication of patients with NPC, recent advances in quantitative magnetic resonance imaging (MRI) sequences, including diffusion-weighted MRI and apparent diffusion coefficient [6,7], dynamic contrast enhancement MRI [7,8], and amide proton transfer imaging [9], have been demonstrated as additional means for the prognostication of patients with untreated NPC. 

Radiomics is the analysis of medical images into high-throughput quantitative data. This field has recently gained significant attention in oncologic radiology research as an illustrative example of personalized precision medicine. The underlying hypothesis is that medical images can reveal important data on tumor phenotypes [10], making radiomics a computational biomarker. One of the benefits of radiomics in medical imaging is its applicability in routinely acquired MRI sequences, such as T2-weighted (T2) or contrast-enhanced T1-weighted (CE-T1) MR images, often yielding thousands of quantifiable imaging features. Zhang et al. investigated the prognostic value of multiparametric MRI-based radiomics for advanced NPC cases [11] and found that MRI-based radiomics provides improved prognostication. The prognostic value of radiomics for untreated NPC has been previously established, further supporting its potential role as a prognostic imaging biomarker [12,13,14]. 

Clarifying the evidence on the role of radiomics in the prognostication of NPC will promote better clinical decision-making for precision medicine. Therefore, the purpose of this systematic review and meta-analysis was to evaluate the prognostic value of MRI-based radiomics for NPC. This study indicated that MRI-based radiomics shows good prognostic performance in predicting the progression-free survival (PFS) of patients with untreated NPC.

## 2. Methods

This study was conducted according to the Preferred Reporting Items for Systematic Reviews and Meta-Analyses (PRISMA) guidelines [15]. This protocol is registered with Open Science Framework (OSF) at https://doi.org/10.17605/OSF.IO/7KADY (accessed date: 17 January 2022).

### 2.1. Literature Search

The PubMed-MEDLINE and EMBASE databases were searched for relevant original articles on the use of MRI-based radiomics for predicting the prognosis of patients with untreated NPC until 12 August 2021. The following search terms were used: [(nasopharyngeal) AND (cancer OR carcinoma OR squamous cell carcinoma OR malignancy OR tumor) AND (MRI OR MR OR magnetic resonance imaging) AND (radiomics OR radiomic OR texture) AND (survival OR prognosis)]. Only articles published in English, and those involving human patients were included. The bibliographies of the selected articles were further screened to identify other potentially relevant articles. 

### 2.2. Inclusion and Exclusion Criteria

The inclusion criteria were as follows, (1) patients: those with pathologically-confirmed NPC without prior treatment, including neoadjuvant chemotherapy, definitive chemoradiation, or radiation therapy; (2) index test: MRI with provision for pretreatment radiomic analysis of primary NPC; (3) reference standard: standards for PFS as determined through clinical/imaging follow-up; and (4) study design: all observational studies (retrospective or prospective).

The exclusion criteria were as follows, (1) case reports, review articles, editorials, letters, and conference abstracts; (2) insufficient data on patients’ survival outcomes; (3) lack of data on the radiomic analysis of primary NPC; (4) insufficient details on patient survival data and Harrell’s concordance index (C-index); and (5) overlapping patient data. Two reviewers (S.L. and Y.C.) independently selected the appropriate study reports using a standardized form. 

### 2.3. Data Extraction

The following data were extracted from the included articles in a standardized format: (a) study characteristics (authors, year of publication, study design, and affiliation); (b) cohort characteristics (number of included patients, including the numbers of the patients in the training and validation cohorts, patients’ mean age, sex, cancer stages, and type of treatment received); (c) MRI protocols (MR pulse sequences used, MR Tesla, manufacturer, and name of scanner); (d) characteristics of radiomic analysis (segmentation software, segmentation method, radiomic software, feature selection method, number of selected radiomic features, use of internal or external validation, and type of algorithm); and (e) model performance metrics (types of models built and their C-indices in the training and validation cohorts). Radiomic models were chosen for analysis for studies in which the C-indices of multiple survival models were reported. 

### 2.4. Quality Assessment Based on the TRIPOD Statement and RQS

Two reviewers (S.L. and Y.C.) independently extracted the data from the articles and performed a quality assessment in consensus. The studies reported in the included articles were evaluated using the Transparent Reporting of a Multivariable Prediction Model for Individual Prognosis or Diagnosis (TRIPOD) checklist, which consists of 22 main criteria with 35 items [16,17]. The type of predictive model was determined as one of the following: development only (type 1a), development and validation using resampling (type 1b), random split-sample validation (type 2a), nonrandom split-sample validation (type 2b), validation using separate data (type 3), or validation only (type 4). To ensure the robustness of the predictive models, only studies of model type 1b or higher were included. Furthermore, studies with less than 50% adherence rate to the TRIPOD checklist items (<18 out of 35 items) were excluded. 

Furthermore, the radiomic characteristics of studies were assessed using the Radiomic Quality Score (RQS). RQS consists of six key domains and measures the robustness of the radiomic methodology by scoring specific points for each category up to a total of 36 points [18]. Scoring of the specific RQS items was based on a previous report [19]. The two reviewers (S.L. and Y.C.) independently evaluated RQS and then in consensus. 

### 2.5. Definitions of Prognostic Endpoints

The definitions of PFS, local relapse-free survival, distant metastasis-free survival, disease-free survival, and failure-free survival were interchangeable among studies; thus, we collectively defined them as PFS: the interval between the first day of treatment to the date of disease progression (either locoregional recurrences or distant metastases), death from any cause, or the date of the last follow-up visit. 

### 2.6. Data Synthesis for Meta-Analysis 

The performances of the radiomics models in predicting PFS, measured using the mean C-indices, were the main outcomes of interest. The C-index measures the prognostic performance of models whose outcomes are time-to-event censored data [20]. Only C-indices calculated from the validation or test datasets were used. For studies in which more than one C-index of radiomic models were reported, the one with the highest C-index was chosen. The 95% confidence intervals (CI) of the associated C-indices were back-calculated to derive their standard deviations (SD) [21]. C-indices calculated from integrated models (i.e., radiomic + clinical or other models) were not used in the analysis. The inverse variance method was used to calculate weights, whereas pooled estimates with their 95% CI were calculated using DerSimonian–Laird random-effects modeling. Between-study heterogeneity was assessed using Q tests and the Higgins inconsistency index (*I*^2^), with *I*^2^ > 50% suggesting heterogeneity [22]. Subgroup meta-regression analyses were performed according to the total number of patients, segmentation method used, number of radiomic features used, external validation, TRIPOD adherence rate, feature selection method, and radiomic software used. Publication bias was assessed using funnel plots and Egger’s test [23]. All statistical analyses were performed using R Statistical Software (version 4.1.0, Vienna, Austria) with ‘metafor’ and ‘meta’ packages [24,25]. 

## 3. Results

### 3.1. Literature Search

**Figure 1:**
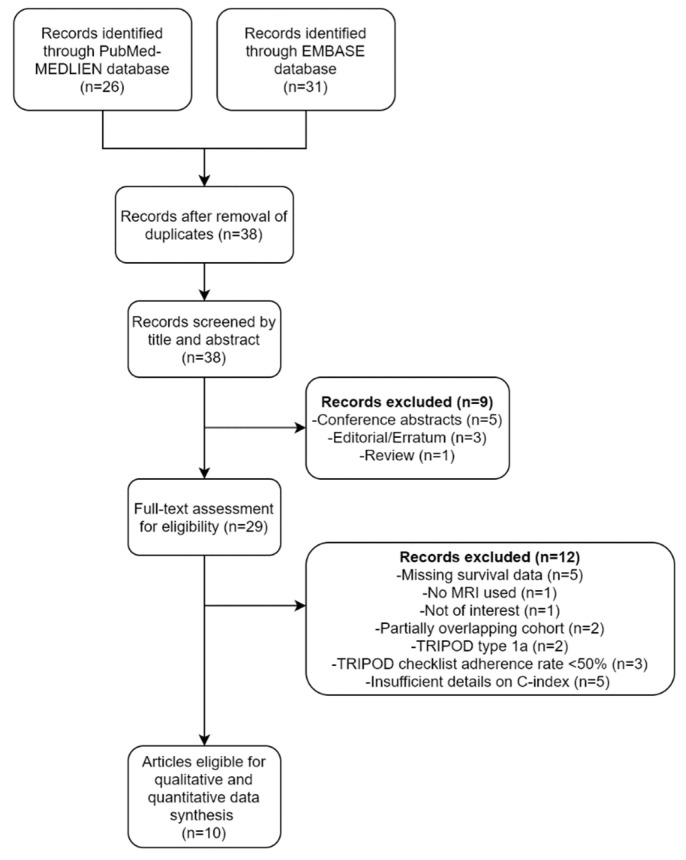
A flow diagram of the study selection process.

A flow diagram of the selection process is depicted in Figure 1. A total of 38 unique articles were screened based on their titles and abstracts. Nine articles, including five conference abstracts, three editorials/errata, and one review, were excluded. The full texts of the remaining 29 articles were then thoroughly reviewed. An additional 12 articles were excluded because the studies reported in them were not conducted using MRI (*n* = 1), not in the field of interest (*n* = 1), were missing survival information (*n* = 5), had partially overlapping cohorts (*n* = 2), or involved the analysis of TRIPOD type 1a predictive models (*n* = 2). Of the two studies with overlapping cohorts, the one with a larger sample size was selected. Finally, 10 studies that met the eligibility criteria were included for data synthesis [11,12,26,27,28,29,30,31,32,33]. 

### 3.2. Clinical Characteristics and MR Protocols of the Included Studies

The detailed characteristics of the 10 eligible studies are summarized in Table 1. While one study was a prospective study [31], the others were retrospective studies. Eight studies were conducted in China [11,27,28,29,30,31,32,33], one in Italy [26] and one in the Republic of Korea [12]. The type of treatment patients received was not reported in two studies [11,27], whereas the types of treatments received, including a combination of radiation, concurrent chemoradiation, induction chemotherapy, or adjuvant chemotherapy, were reported in the other studies. The 1.5 Tesla and 3.0 Tesla MR scanners were used in six [11,26,27,28,29,31] and two studies [12,33], respectively, whereas both the 1.5 and 3.0 Tesla MR scanners were used in two studies [30,32]. T2 and CE-T1 sequences were used in all but two studies, in which only CE-T1 [27] and T2 with non-contrast T1 sequences [26] were used.

### 3.3. Radiomic and Image Analyses

The details of the radiomic and image analyses of the included studies are summarized in Table 2. Regarding the selection of the region of interest, whole tumors were segmented in seven studies [11,12,28,29,30,32,33], whereas only the largest axial slice was segmented in two studies [26,31]. However, the segmentation method used was not reported in one study [27]. Regarding feature selection methods, the least absolute shrinkage and selection operator (LASSO) was used for feature selection in six studies [11,12,27,28,29,32], whereas recursive feature elimination [31], stability and correlation-based selection [26], and minimal redundancy maximum relevance with random forest [30], were used in the other studies. Except for one study in which feature selection was not applied [33], the number of radiomic features selected for the prognostic models analyzed in the other studies ranged from two to 20. Both internal and external validation of the models were performed in only two studies [30,31]. All studies used machine learning algorithms for the radiomic analysis except for two studies that used the deep learning [32] and conventional statistical methods [26]. 

### 3.4. Quality Assessment of the Prediction Models Based on the TRIPOD Statement

Among the 35 items of the TRIPOD checklist, the mean ± SD of the reported TRIPOD items was 24 ± 2.5. The mean adherence rate and SD of the checklist was 68.6 ± 7.1%. Most importantly, none of the studies presented their titles as ‘developing/validating a model, target population, and the outcome’. Moreover, none of the articles described the handling of data or details of any imputation method. The checklist of the individual TRIPOD items is summarized in Supplementary Table S1. 

The basic adherence rate of RQS items is summarized in Table 3. The mean adherence rate was 55 ± 43%. All studies included validation cohorts and conducted a cut-off analysis (i.e., determining high- and low-risk groups) and discrimination statistics (i.e., reporting C-index with 95% CI). None of the studies adhered to the RQS items in Domain 5 and 6. The detailed scores of each item are provided in Supplementary Table S2. 

### 3.5. Pooled Estimate of C-Indices for PFS

**Figure 2:**
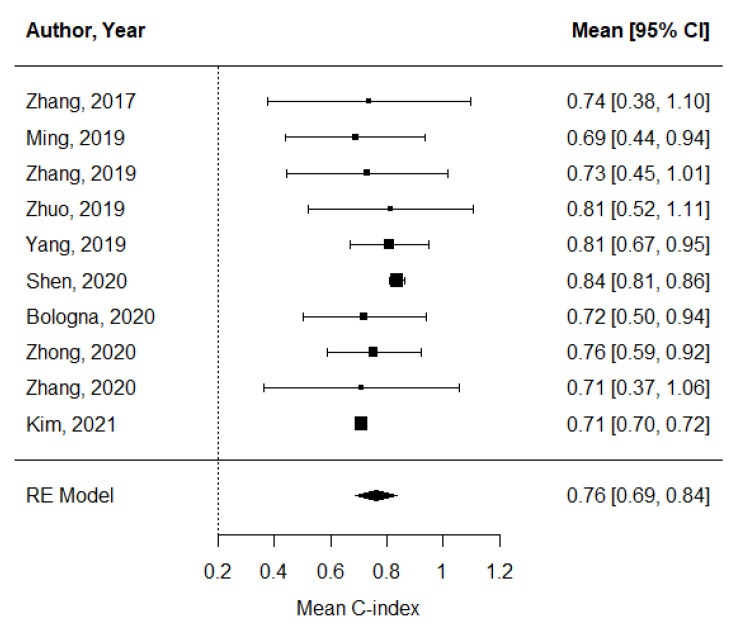
A forest plot of the pooled estimate of C-indices of progression-free survival.

**Figure 3:**
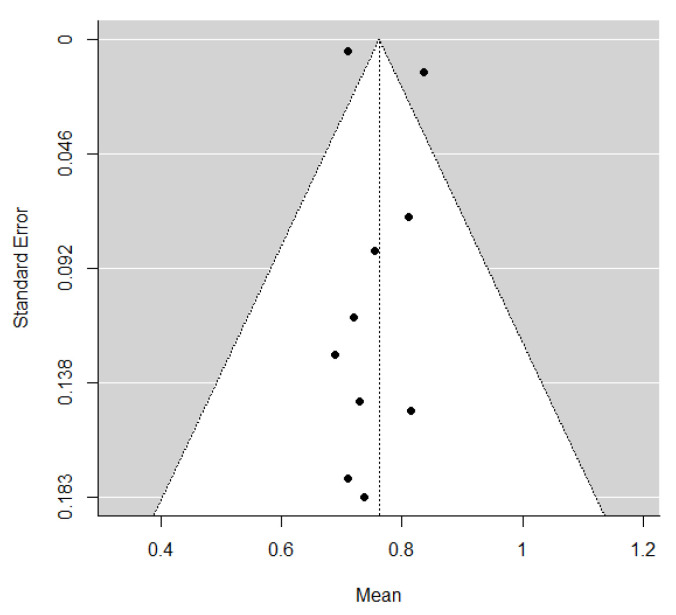
A funnel plot of C-indices.

The pooled estimate of C-indices for PFS was 0.76 (95% CI, 0.69–0.84) (Figure 2). In addition, there was significant heterogeneity among the studies (*I*^2^ = 89.2%; Cochran’s Q method, *p* < 0.001). No publication bias was observed upon visual inspection of the funnel plot (Figure 3); the results of Egger’s test showed no bias as well (*p* = 0.73). 

### 3.6. Subgroup Meta-Regression Analyses

The results of the subgroup meta-regression analysis are shown in Table 4. The number of radiomic features used in the prognostic models and the radiomic software used were found to be the sources of heterogeneity. Models developed using more than eight radiomic features had significantly higher C-indices than those developed with less features (0.83 vs. 0.71, *p* < 0.001). Furthermore, models developed using PyRadiomics for radiomic feature extraction had significantly lower C-indices than those developed using other software (0.71 vs. 0.83, *p* < 0.001). Other covariates, including the segmentation method used (whole tumor vs. largest axial slice, *p* = 0.53), total number of patients (>300 vs. ≤300, *p* = 0.686), external validation of the prognostic models (yes vs. no, *p* = 0.542), rate of adherence to the TRIPOD checklist (>70% vs. ≤70%, *p* = 0.775), and feature selection method (LASSO vs. others, *p* = 0.975) were not found to be significant sources of heterogeneity. One article [27] did not specify whether the ROI segmentation performed in the reported study was based on the largest axial slice or whole tumor; thus, the study was not included in the subgroup meta-regression analysis of the segmentation method covariate. 

## 4. Discussion

This systematic review and meta-analysis were conducted to assess the prognostic value of pretreatment MRI-based radiomics for NPC. Based on the pooled estimate of the C-indices of the analyzed models, radiomics revealed an overall modest prognostic value in predicting PFS (mean C-index, 0.76; 95% CI, 0.69–0.84). However, there was substantial heterogeneity across the studies, which was primarily due to the number of radiomic features included in the prognostic models. 

Of the 10 selected studies, the study by Shen et al. reported the highest C-index (0.84; 95% CI, 0.64–0.89) [28]. A possible explanation for this might be that except for the study in which feature selection was not performed, the prognostic model in the study by Shen et al. had the largest number of radiomic features (*n* = 20) [33]. This finding is in line with that of our subgroup meta-regression analysis, which showed that the number of radiomic features was a significant factor in determining the performance of the C-index. This is also consistent with the results of a previous study by Chu et al. which indicated that a larger number of radiomic features is more accurate than a lower number in discriminating pancreatic ductal adenocarcinoma from the normal pancreas [34]. However, it is important to emphasize that radiomic models fitted with a larger number of features are also more susceptible to overfitting, which in turn inevitably impacts the reproducibility in external datasets. In this regard, the two studies that reported C-indices of 0.73 and 0.71 obtained from the external validation cohorts [30,31] may provide higher clinical values than other studies with internal validation cohorts. Of note, the study by Zhang et al. [31] applied the harmonization of MR images to correct for inter-scanner variabilities, which is particularly important for standardizing radiomic features obtained from different MRI scanners. 

Interestingly, between-study heterogeneity in the subgroup meta-regression analysis was attributable to the software used for radiomic feature extraction. The models developed using PyRadiomics for radiomic feature extraction demonstrated significantly lower C-indices than those developed using other software. Considering that PyRadiomics is a rigorously tested and maintained software that serves as a reference standard for radiomic analysis [35], it seems counterintuitive that models designed using PyRadiomics showed lower C-indices than those designed using other software. A possible interpretation of this finding is that PyRadiomics-derived radiomic features are more standardized with a relatively smaller number of radiomic features to choose from (around 120 features). However, handcrafted radiomic features acquired using MATLAB are easier to use for additional specifications such as wavelet filter application and log transformations, thus yielding a substantially larger pool of radiomic features. We investigated other possible factors specific to radiomic research that may be responsible for between-study heterogeneity, such as the method of feature reduction or external validation of prognostic models, but none of them yielded meaningful findings. 

Despite the benefits of radiomics in oncologic imaging, its applicability in routine medical imaging without the need for additional advanced time-consuming MRI protocols is limited: a frequent criticism on radiomic research focuses on the lack of reproducibility of radiomic features and generalizability of clinical settings [36]. Among the included studies, many selected radiomic features were highly handcrafted such that none of the features within the same broad category (i.e., gray-level co-occurrence matrix) had any meaningful overlap when they were further subcategorized after the wavelet filter application, which potentially limits the repeatability of the research. One plausible explanation for this finding is the lack of preprocessing (i.e., histogram matching or z-score normalization) of features extracted from MRI sequences in half of the selected studies [11,27,28,29,33]. Image preprocessing and normalization are especially relevant in the context of MRI scans, where the absolute voxel intensities do not have tissue-specific meanings [37].

It is notable that the mechanism by which specific radiomic features’ characteristics may translate into patient prognosis was not reported in several of the studies [28,31,32]. This may lead to limited reproducibility and repeatability of radiomic research. The selected features of importance varied greatly among studies, even among those where the same two sequences (i.e., T2 and T1-CE) were used for feature extraction [11,12,28,29]. This suggests that the extraction of radiomic features is highly data-dependent and susceptible to variations in manual segmentations [38]. 

It is also interesting to note that some of the criteria of the TRIPOD checklist were not met by all studies. For instance, none of the study titles indicated the target population of the study, the outcome of the study, or whether the studies were conducted to develop or validate a model. This is consistent with the findings of a recent study on the quality of reporting radiomics in oncologic studies according to the TRIPOD statement [17]. Among the 77 studies reviewed in that study, only the titles of two studies were in line with the TRIPOD recommendations. Similarly, another study by Heus et al. showed that appropriate titles were the least well-reported items [39]. This may lead to difficulties in identifying published studies on prediction models. As for the RQS assessment, none of the studies met items such as phantom study or multiple imaging acquisition. In retrospective study design, adhering to such items would probably be challenging in clinical settings. Overall, the RQS of the included studies was unsatisfactory with all scores below 50%, which is consistent with other similar systematic reviews based on the RQS [40,41,42]. 

This study has some limitations. First, a pooled estimate of overall survival could not be calculated because of the small number of studies with overall survival as the primary endpoint. Second, most of the included studies were conducted in China because NPC is endemic in southern China. Thus, the geographically imbalanced data may limit the generalizability of our findings. Finally, only prognostic models fitted with radiomic features were assessed because clinical factors, and prognostic clinical models, were highly variable across the studies, and thus were not suitable for calculating pooled estimates. 

## 5. Conclusions

The findings of the present study suggest that pretreatment MRI-based radiomics has a prognostic value in predicting the PFS of patients with NPC. The subgroup meta-regression analysis showed that the number of radiomic features selected in the prognostic models is significantly associated with C-index performance. However, there was substantial heterogeneity across the studies; thus, more consistent and robust study protocols are necessary in future radiomics research.

## Figures and Tables

**Table 1:** Clinical characteristics and magnetic resonance (MR) protocols of the included studies.

First Author (Year of Publication)	Affiliation and Country	Study Period	Study Design	No. of Patients (Training/Validation)	Age [Mean ± SD or Median (Range)]	Proportion of Male	Overall and TNM Cancer Stage	Type of Treatment Received	MR Tesla	MR Pulse Sequences	MR Manufacturer (Scanner Name)
Zhang B (2017)	Guangdong General Hospital/Guangdong Academy of Medical Sciences, China	January 2007–August 2013	Retrospective	118 (88/30)	43 (38–51) (training)/ 44 (36–51) (validation)	78%	Non-metastatic III–IVa	NR	1.5	T2, CE-T1	GE (Signa EXCITE HD, TwinSpeed)
Ming X (2019)	Fudan University Shanghai Cancer Center, China	January 2010–February 2012	Retrospective	303 (200/103)	48 (11–80)	74.6%	I–IV	NR	1.5	CE-T1	GE
Zhang L (2019)	Sun Yat-sen University Cancer Center, China	April 2009–December 2015	Prospective	737 [360/120(internal)/ 257(external)]	NR ^1^	75%	Non-metastatic I–IVa	RT alone or CCRT ± IC ± AC	1.5	T1, T2, CE-T1	GE (Signa EXCITE, SignaHDx), Siemens (Espree, Novus15)
Zhuo E (2019)	South China University of Technology, China	January 2010–January 2013	Retrospective	658 (424/234)	45 (38–53) (training) 44 (37–50) (validation)	73.3%	Non-metastatic I–IVa	Radical IMRT	3	T1, T2, CE-T1	GE (Discovery MR750)
Yang K (2019)	Cancer Center and State Key Laboratory of Biotherapy, West China Hospital, Sichuan University, China	January 2010–February 2013	Retrospective	224 (149/75)	46 ± 11 (training) 50 ± 10 (validation)	70.1%	III–IVa	RT ± IC ± AC	1.5	T2, CE-T1	Siemens (TrioTrim)
Shen H (2020)	Chongqing University Cancer Hospital and Chongqing Cancer Institute and Chongqing Cancer Hospital, China	June 2013–June 2017	Retrospective	327 (230/97)	52 (45–61) (training) 52 (45–61) (validation)	72.5%	Non-metastatic I–IVa	RT alone or CCRT ± IC ± AC	1.5	T2, CE-T1	Philips (Achieva)
Bologna M (2020)	Fondazione IRCCS Istituto Nazionale dei Tumori, Italy	2004–2017	Retrospective	136	48 (39–57)	70%	I–IV	RT alone or CCRT ± IC	1.5	T1, T2	Siemens (Magnetom Avanto)
Zhong L (2020)	School of Artificial Intelligence, University of Chinese Academy of Sciences, China	January 2010–March 2016	Retrospective	638 (447/191)	41 (10–69) (training) 41 (16–68) (validation)	69.3%	Non-metastaticI–III	IC + CCRT	1.0, 1.5, 3.0	T1, T2, CE-T1	Philips (Achieva, Panorama HFO) GE (Discovery MR750, Espree, Signa EXCITE, Signa HDx), Siemens (TrioTim)
Zhang F (2020)	The Cancer Center of the Fifth Affiliated Hospital, Sun Yat-sen University, China	January 2013–November 2019	Retrospective	236 [132/44(internal) /44(external)]	48 (19–83) (training) 49 (27–78) (internal test) 44 (24–70) (external test)	72.7%	Non-metastaticI–IVa	RT	1.5, 3	T1, T2, CE-T1	Siemens (Magnetom Verio, Avanto)
Kim M (2021)	Seoul St. Mary’s Hospital, College of Medicine, The Catholic University of Korea, Republic of Korea	June 2006–October 2019	Retrospective	81 (57/24)	53 ± 13	75.3%	Non-metastaticI–IVa	CCRT	3	T2, CE-T1	Siemens (Magnetom Verio)Philips (Ingenia)

^1^ Reported as <62 or ≥62 years old. AC = adjuvant chemotherapy; CCRT = concurrent chemoradiation therapy; CE-T1 = contrast-enhanced T1-weighted image; IC = induction chemotherapy; IMRT = intensity-modulated radiation therapy; NA = not available; NR = not reported; SD = standard deviation.

**Table 2:** Summary of details of radiomic and image analyses.

First Author (Year of Publication)	Segmentation Software	Segmentation Method	Radiomic Software Used	Feature Selection Method	Number of Radiomic Features Selected	Validation Type	Type of Algorithm Used
Zhang B (2017)	ITK-SNAP	Whole tumor	MATLAB	LASSO	8	I	ML
Ming X (2019)	MIM	Not reported	MATLAB	LASSO	5	I	ML
Zhang L (2019)	RadiAnt	Largest axial slice	MATLAB	Recursive feature elimination	11	I & E	ML
Zhuo E (2019)	Analyze Pro	Whole tumor	MATLAB	None	4863	I	ML
Yang K (2019)	Raystation	Whole tumor	LIFEx	LASSO	3	I	ML
Shen H (2020)	In-house software developed by Philips	Whole tumor	Philips Radiomics Tool	LASSO	20	I	ML
Bologna M (2020)	Not reported	Largest axial slice	PyRadiomics	Stability-based selection, correlation-based selection	2	I	Statistical method
Zhong L (2020)	ITK-SNAP	Whole tumor	PyRadiomics	LASSO	3	I	DL
Zhang F (2020)	ITK-SNAP	Whole tumor	PyRadiomics	ICC, minimal redundancy maximum relevance, random forest	12	I & E	ML
Kim M (2021)	3D Slicer	Whole tumor	PyRadiomics	LASSO	7	I	ML

DL = deep learning; E = external validation; I = internal validation; LASSO = least absolute shrinkage and selection operator; ML = machine learning.

**Table 3:** Basic adherence rate of the RQS items.

RQS items	Adherence Rate
Domain 1	
Image protocol quality	90% (9)
Multiple segmentation	20% (2)
Phantom study on all scanners	0%
Imaging at multiple time points	0%
Domain 2	
Feature reduction or adjustment for multiple testing	90% (9)
Validation	100% (10)
Domain 3	
Multivariate analysis with non-radiomics features	90% (9)
Detect and discuss biologic correlates	60% (6)
Comparison to gold standard	90% (9)
Potential clinical utility	70% (7)
Domain 4	
Cut-off analysis	100% (10)
Discrimination statistics	100% (10)
Calibration statistics	70% (7)
Domain 5	
Prospective study registered in a trial database	0%
Cost-effective analysis	0%
Domain 6	
Open science and data	0%

**Table 4:** Subgroup meta-regression analysis of included studies.

Covariate	No. of Studies	C-Index (95% CI)	*p*-Value ^1^
No. of patients			
>300	5	0.76 (0.60–0.92)	0.686
≤300	5	0.74 (0.68–0.78)	
Segmentation method ^2^			
Whole tumor	7	0.75 (0.65–0.85)	0.53
Largest axial slice	2	0.72 (0.66–0.79)	
No. of radiomic features used			
<8	5	0.71 (0.70–0.73)	<0.001
≥8	5	0.83 (0.81–0.86)	
External validation			
Yes	2	0.72 (0.60–0.84)	0.542
No	8	0.74 (0.66–0.83)	
TRIPOD adherence rate			
>70%	6	0.75 (0.63–0.86)	0.775
≤70%	4	0.73 (0.65–0.82)	
Feature selection method			
LASSO	6	0.74 (0.63–0.85)	0.975
Others	4	0.74 (0.67–0.82)	
Radiomic software			
PyRadiomics	4	0.71 (0.69–0.73)	<0.001
Others	6	0.83 (0.81–0.85)	

^1^*p*-value for between-group difference according to each category. ^2^ One study (Ming et al) did not specify the segmentation method and was not included in the subgroup meta-regression analysis. LASSO = least absolute shrinkage and selection operator; TRIPOD = Transparent Reporting of a Multivariable Prediction Model for Individual Prognosis or Diagnosis.

## Supplementary Materials

The following supporting information can be downloaded at: https://www.mdpi.com/article/10.3390/cancers14030653/s1, Table S1: Adherence rate of the TRIPOD items of the included studies; Table S2: Details of radiomic quality score.
