# Supplementary material for: Magnetic Resonance Imaging-Based Radiomics for the Prediction of Progression-Free Survival in Patients with Nasopharyngeal Carcinoma: A Systematic Review and Meta-Analysis
Source: Cancers (Basel). 2022 Jan 27;14(3):653. doi: 10.3390/cancers14030653 (PMC8833585; doi:10.3390/cancers14030653)
Supplement: Supplementary file 1 [file cancers-14-00653-s001.zip › TableS1.pdf]

Table S1. Adherence rate of the TRIPOD items of the included studies

	All studies (n = 10)
**Total (35 items)**	24 (68.6%)
**Title and abstract**	
1. Title: identify developing/validating a model, target population, and the outcome	0 (0)
2. Abstract: provide a summary of objectives, study design, setting, participants, sample size, predictors, outcome, statistical analysis, results, and conclusions	10 (100)
**Introduction**	
3a. Explain the medical context and rationale for developing/validating the model	10 (100)
3b. Specify the objectives, including whether the study describes the development/validation of the model or both	7 (70)
**Methods**	
4a. Source of data: describe the study design or source of data (randomized trial, cohort, or registry data)	10 (100)
4b. Source of data: specify the key dates	10 (100)
5a. Participants: specify key elements of the study setting including number and location of centers	8 (80)
5b. Participants: describe eligibility criteria for participants (inclusion and exclusion criteria)	10 (100)
5c. Participants: give details of treatment received, if relevant (n = 12)	5 (50)
6a. Outcome: clearly define the outcome, including how and when assessed	9 (90)
6b. Outcome: report any actions to blind assessment of the outcome	0 (0)
7a. Predictors: clearly define all predictors, including how and when assessed	6 (60)
7b. Predictors: report any actions to blind assessment of predictors for the outcome and other predictors	0 (0)
8. Sample size: explain how the study size was arrived at	1 (10)
9. Missing data: describe how missing data were handled with details of any imputation method	0 (0)
10a. Statistical analysis methods: describe how predictors were handled	
10b. Statistical analysis methods: specify type of model, all model-building procedures (any	10 (100)
predictor selection), and method for internal validation	10 (100)
10d. Statistical analysis methods: specify all measures used to assess model performance and if	
relevant, to compare multiple models (discrimination and calibration)	9 (90)
11. Risk groups: provide details on how risk groups were created, if done (yes or no, n = 77)	
**Results**	9 (90)
13a. Participants: describe the flow of participants, including the number of participants with	
and without the outcome. A diagram may be helpful	5 (50)
13b. Participants: describe the characteristics of the participants, including the number of	

participants with missing data for predictors and outcome	9 (90)
14a. Model development: specify the number of participants and outcome events in each analysis	
14b. Model development: report the unadjusted association between each candidate predictor and outcome, if done (yes or no, n = 77)	7 (70)
15a. Model specification: present the full prediction model to allow predictions for individuals (regression coefficients, intercept)	6 (60)
15b. Model specification: explain how to the use the prediction model (nomogram, calculator, etc)	7 (70)
16. Model performance: report performance measures (with confidence intervals) for the prediction model	9 (90)
**Discussion**	9 (90)
18. Limitations: Discuss any limitations of the study	
19b. Interpretation: Give an overall interpretation of the results	
20. Implications: Discuss the potential clinical use of the model and implications for future	10 (100)
research	10 (100)
**For validation (types 2a, 2b, 3, and 4) n = 54**	10 (100)
10c. Methods-Statistical analysis methods: describe how the predictions were calculated	
10e. Methods-Statistical analysis methods: describe any model updating (recalibration), if done	
12. Methods-Identify any differences from the development data in setting, eligibility criteria,	9 (90)
outcome, and predictors	(-)
13c. Results-show a comparison with the development data of the distribution of important variables	8(80)
17. Results-Model updating: report the results from any model updating, if done	8 (80)
19a. Discussion-Interpretation: discuss the results with reference to performance in the	
development data and any other validation data	(-)
	9 (90)
