# Supplementary material for: Magnetic Resonance Imaging-Based Radiomics for the Prediction of Progression-Free Survival in Patients with Nasopharyngeal Carcinoma: A Systematic Review and Meta-Analysis
Source: Cancers (Basel). 2022 Jan 27;14(3):653. doi: 10.3390/cancers14030653 (PMC8833585; doi:10.3390/cancers14030653)
Supplement: Supplementary file 1 [file cancers-14-00653-s001.zip › TableS2_RQS.pdf]

Table S2. Details of radiomic quality score

**First author**	**Domain 1**				**Domain 2**		**Domain 3**				**Domain 4**			**Domain 5**		**Domain 6**	
	Image protocol quality	Multiple segmentations	Phantom study on all scanners	Imaging at multiple time points	Feature reduction or adjustment for multiple testing	Validation	Multivariable analysis with non-radiomics features	Detect and discuss biological correlates	Comparison to 'gold standard'	Potential clinical utility	Cut-off analyses	Discrimination statistics	Calibration statistics	Prospective study registered in a trial database	Cost-effectiveness analysis	Open science and data	Total
Zhang B (2017)	1	0	0	0	3	2	1	1	2	2	1	1	1	0	0	0	15 (41.7%)
Ming X (2019)	1	1	0	0	3	2	1	1	2	0	1	1	0	0	0	0	13 (36.1%)
Zhang L (2019)	1	0	0	0	3	4	1	0	2	2	1	1	1	0	0	0	16 (44.4%)
Zhuo E (2019)	1	0	0	0	0	2	0	0	0	0	1	1	0	0	0	0	5 (13.9%)
Yang K (2019)	1	0	0	0	3	2	1	1	2	2	1	2	1	0	0	0	16 (44.4%)
Shen H (2020)	1	1	0	0	3	2	1	0	2	2	1	1	1	0	0	0	15 (41.7%)
Bologna M (2020)	0	0	0	0	3	2	1	1	2	0	1	1	0	0	0	0	11 (30.6%)
Zhong L (2020)	1	0	0	0	3	2	1	0	2	2	1	1	1	0	0	0	14 (38.9%)
Zhang F (2020)	1	0	0	0	3	4	1	1	2	2	1	1	1	0	0	0	17 (47.2%)
Kim M (2021)	1	0	0	0	3	2	1	1	2	2	1	1	1	0	0	0	15 (41.7%)
